# Boosting liver regeneration: kinase inhibitor as a new tool to prevent liver failure

**DOI:** 10.1038/s41392-024-01879-0

**Published:** 2024-07-03

**Authors:** Anna Sichler, Norbert Hüser, Klaus-Peter Janssen

**Affiliations:** Department of Surgery, School of Medicine and Health, Technical University of Munich, Munich, Germany

**Keywords:** Drug development, Gastrointestinal diseases, Structural biology, Metastasis

In a recent manuscript in *Cell*, a first-in-class small molecule inhibitor of the kinase MKK4 (mitogen-activated protein kinase kinase 4) was described that greatly enhances liver regeneration after partial hepatectomy in animal models, with an excellent safety profile in humans.^1^ The findings of Zwirner et al. hold promise to alter the clinical management of liver failure in critically ill patients.^1^

Liver disease is one of the world’s global health challenges; it can be induced by viral infections, toxins, or metabolic stress leading to fibrosis, cirrhosis, alcoholic steatohepatitis (ASH), and metabolic dysfunction associated steatohepatitis (formerly known as nonalcoholic steatohepatitis (NASH)).^2^ Moreover, primary hepatic malignancies or metastasis of colorectal and other cancers can affect the liver.^2^ The common treatment is partial hepatectomy (PHx), based on surgical removal of the cancer-affected tissue. Assessment of the outcome can be challenging due to heterogenous pathologies and interventions, but 5-year survival rates around 50% were reported.^2^ A radical, oncologically adequate procedure is only possible due to the extraordinary regenerative capacity of the adult liver, illustrated by the fact that up to 75% of healthy liver can be safely removed. However, the processes that govern liver regeneration are not fully understood, and regenerative capacities are drastically limited under disease conditions.

**Fig. 1:**
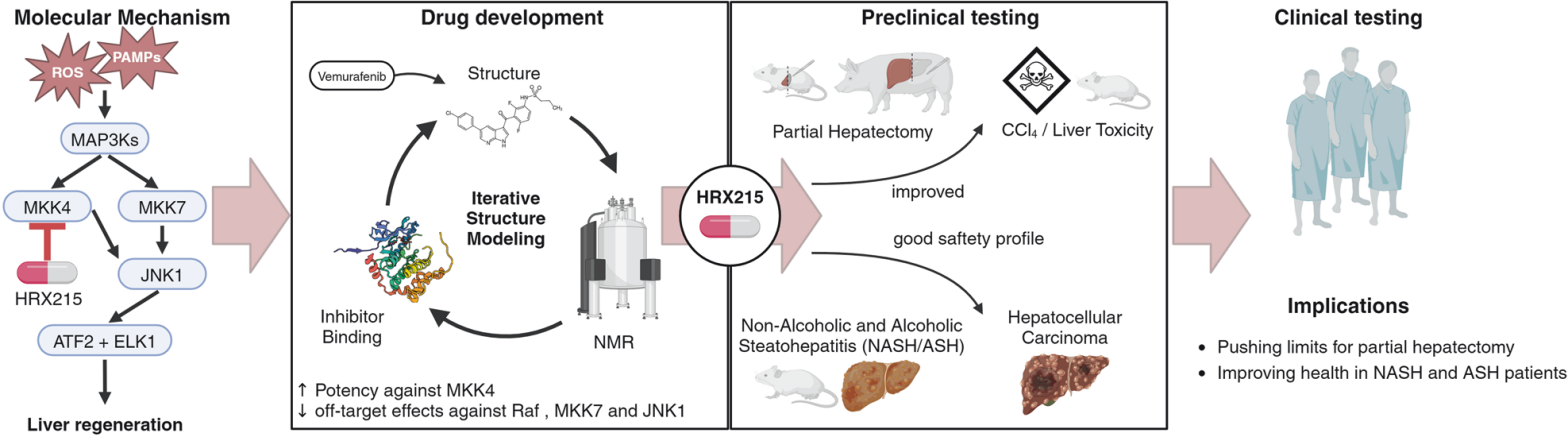
The new compound (HRX215) inhibits MKK4 and increases hepatocyte survival and liver regeneration. Left, cell stress caused by, e.g., reactive oxygen species (ROS) or pathogen-associated molecular patterns (PAMPs) induces the kinases MKK4 and MKK7. Inhibition of MKK4 by the new compound HRX215 leads to activation of proliferation-enhancing pathways downstream of MKK7 and JNK1. The small molecule compound was developed in an iterative fashion, based on NMR-enabled structure modeling, and then tested on small and large animal models. It increased survival after partial hepatectomy and showed antifibrotic and antisteatotic activity, was displaying excellent pharmacological safety in a phase I study, and has no adverse effects such as tumor formation. Created with BioRender.com

Whereas many studies investigated liver regeneration, only few approaches have been translated into the clinic. Zwirner et al. placed their focus on the MAP kinase family. Currently, MAPK family inhibitors are studied for the treatment of multiple diseases.^3^ Among these kinases, central signaling hubs in eukaryotic cells, MKK4 stands out as stress-response factor and promising target in hepatocytes. The mechanism of action of MKK4 has been investigated by the same researchers previously.^4^ MKK4 is induced in hepatocytes under stress conditions, such as partial hepatectomy, chemically induced damage or steatohepatitis. By genetic deletion or RNA interference of MKK4 in mouse models, the anti-proliferative effects of MKK4 were blocked, whereas the kinase MKK7 was still activated, leading downstream via JNK1 to activation of the transcription factors ATF2 and ELK1, and ultimately to enhanced proliferation and liver regeneration (Fig. 1). MKK4 can be activated by innate Toll-like receptor signaling, the question therefore arises if microbial derived factors play a role in the MKK4-mediated effects, given the presumed role of the microbiome in liver regeneration.^5^

The authors opted for a small molecule approach and were able to develop a novel “first-in-class” inhibitor for MKK4. This compound (HRX215) reduces hepatocyte cell death after damage, shows antifibrotic and antisteatotic effects and, importantly, is well tolerated and does not induce tumorigenesis in the liver. The authors selected a small molecule-based compound with known off-target effects on MKK4, the BRAF^V600E^ inhibitor Vemurafenib. In a nuclear magnetic resonance (NMR) spectroscopy based approach, based on already known structures, the potency and selectivity against MKK4 was improved in a stepwise fashion, while removing affinity to Raf kinases, MKK7 and JNK1. Further, the bioavailability, metabolic clearance and membrane permeability were considered during this process.

The authors thoroughly tested the inhibitor in several disease conditions. Hepatocyte proliferation after PHx was substantially enhanced in mouse models, even under fibrotic conditions. In a lethal hepatectomy-induced liver failure model with 85% resection in pigs, HRX215 was able to prevent acute liver failure. Hepatocyte cell death after liver damage induced by carbon tetrachloride in mice was reduced upon HRX215 treatment. Besides, steatosis and fibrosis in ASH and NASH mouse models were reduced under HRX215 treatment, indicating antisteatotic and antifibrotic activity. Importantly, long-term treatment did not induce unwanted side effects in other tissues, and did not increase tumorigenesis in a liver cancer model (GAN-NASH diet). Generally, treatment with HRX215 seems to be well tolerated, since MKK4 only limits hepatocyte proliferation under stress conditions. The authors also tested the effects of HRX215 in hepatocellular carcinoma cells. In contrast to its effects in non-malignant hepatocytes, the MKK4 inhibitor decreased proliferation, possibly due to activation of p38 and suppression of the cell cycle regulator CDC25C.

Finally, safety of HRX215 was tested following good laboratory practice protocols in rodents and non-rodent models, and in a clinical phase I trial. Remarkably, HRX215 showed good pharmacological availability, with no severe or serious adverse effects, and mild or moderate adverse effects were comparable to the placebo.

This manuscript is a showcase for successful interdisciplinary and translational academic research that lives up to the often heard, but rarely achieved catch phrase “from bench to bedside.” One of the notable strengths lie in the integration of drug development and molecular modeling techniques for the optimization of HRX215 structure. Moreover, rigorous testing of HRX215 in a variety of preclinical models demonstrates its broad applicability and efficacy in diverse clinically relevant scenarios.

However, some questions remain to be answered. First and foremost, clinical trials are warranted to test the pro-regenerative potential of this new compound. Clinically, it is imperative in many cases to increase the volume of the future liver remnant in order to prevent postsurgical liver failure. This can be achieved by enhancing hepatocyte proliferation prior or after hepatectomy by medication. However, the extent to which perioperative or even postoperative administration leads to a rapid and function-preserving effect would be a clinically important question. Portal vein ligation for staged hepatectomy liver partition or portal vein embolization are well-established procedures. However, these methods are sometimes not sufficient to induce a strong proliferation in hepatocytes due to underlying disease, advanced age of patients, or methodological issues. Whether HRX215 would be able to activate hepatocyte proliferation under such adverse conditions also remains to be shown.

Since the compound does not reduce inflammatory processes, it will be needed to be combined with other treatments, and its compatibility remains to be clarified. Further, the effect of HRX215 in virus induced liver damage is unclear. More studies are clearly needed to test whether bypassing the restrictions for living donor transplantation, as well as the issue of “too small for size” are feasible and safe in a scenario with HRX215 treatment. Taken together, the new MKK4 inhibitor holds the promise to transform liver disease management and shows the value of translational research in addressing unmet medical needs.
